# *Cryptosporidium* and cryptosporidiosis: An update of Asian perspectives in humans, water and food, 2015–2025

**DOI:** 10.1016/j.crpvbd.2025.100311

**Published:** 2025-08-22

**Authors:** Shahira Abdelaziz Ali Ahmed, Sonia Boughattas, Mohammad Reza Mahmoudi, Huma Khan, Simuzar Mamedova, Ardra Namboodiri, Frederick R. Masangkay, Panagiotis Karanis

**Affiliations:** a Department of Parasitology, Faculty of Medicine, Suez Canal University, Ismailia, 41522, Egypt; b Biomedical Research Center, Qatar University, PO Box 2713, Doha, Qatar; c Biomedicine Research Center, Trauma Institute, Guilan University of Medical Sciences, Rasht, Iran; d Department of Parasitology and Mycology, School of Medicine, Guilan University of Medical Sciences, Rasht, Iran; e Department of Microbiology, University of Swabi, Khyber Pakhtunkhwa, 23340, Pakistan; f Institute of Zoology, Ministry of Science and Education, Republic of Azerbaijan, Baku, 1001, Azerbaijan; g Department of Life Sciences, Khazar University, Baku, 1001, Azerbaijan; h Department of Science, Faculty of Health and Environmental Sciences, Auckland University of Technology, Auckland Central, 1010, New Zealand; i Department of Medical Technology, Faculty of Pharmacy, University of Santo Tomas, Manila, 1008, Philippines; j Research Center for the Natural and Applied Sciences, University of Santo Tomas, Manila, 1008, Philippines; k Medical Faculty and University Hospital, University of Cologne, Cologne, Germany; l University of Nicosia Medical School, 24005, CY-1700, Nicosia, Cyprus

**Keywords:** *Cryptosporidium*, Cryptosporidiosis, Asia, Genotype, Oocysts, Prevalence

## Abstract

*Cryptosporidium* species are increasingly recognized as significant enteric pathogens, particularly within developing nations, where they pose serious public health challenges. This systematic review and meta-analysis examine a decade of research (2015–2025) to map the epidemiological footprint of *Cryptosporidium* across Asia, incorporating 228 studies from 28 countries and analyzing a collective sample of 327,783 specimens collected from humans, water, and food. The overall pooled prevalence was 8.1%, with Southeast Asia emerging as the region of highest concern. Among the affected populations, immunocompromised individuals and children demonstrated the highest vulnerability. Environmental contamination was especially pronounced in surface water sources, while vegetables, particularly those sold in wholesale markets, were the most contaminated food matrices. Molecular findings identified 23 distinct species, and several subtypes predominated by *C. parvum* (IIa, IId) and *C. hominis* (Ia, Ib). Notably, some water samples from mixed and surface water sources exhibited extraordinarily high oocyst concentrations, reaching up to 80,000 oocysts/l. Diagnostic approaches varied widely, with a considerable proportion of studies employing traditional non-molecular techniques, thereby highlighting the need for more advanced and standardized detection protocols. Despite regional disparities and methodological variability, the findings reveal a consistent pattern of widespread exposure and environmental circulation of *Cryptosporidium* species across the continent. This underscores an urgent need for multisectoral collaborations and interventions aimed at bolstering water and food safety infrastructure, enhancing diagnostic capacity, and strengthening public health systems to effectively manage and prevent cryptosporidiosis throughout Asia.

## Highlights

• Across Asia, *Cryptosporidium* spp. circulated with a prevalence of 8.1%.
• Immuno-compromised individuals and children were the most affected population.
• Surface water had the highest *Cryptosporidium* spp. contamination rate (20.3%).
• Food matrices like fruits/vegetables (wholesale markets) had a contamination rate of 5.6%.
• A total of 23 *Cryptosporidium* species were reported: *C. parvum* and *C. hominis* were dominant.

## Graphical abstract

**Figure:**
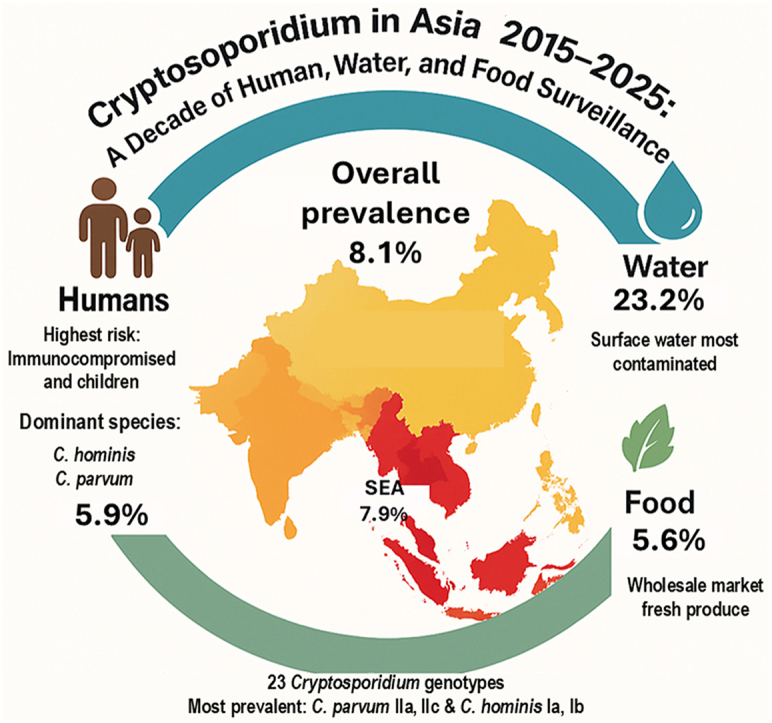

## 1 Introduction

Species of *Cryptosporidium* are increasingly recognized as significant enteric pathogens. The genus was first discovered over a century ago by Tyzzer, who described *Cryptosporidium muris* in the gastric epithelium of laboratory mice (Tyzzer, 1907). *Cryptosporidium* spp. were not recognized as infectious agents in humans until 1976, when the first report of infection with *Cryptosporidium* in a human, a 3-year-old child suffering from severe acute enterocolitis, was published (Nime et al., 1976). *Cryptosporidium* spp. are particularly prevalent in low-to middle-income countries, and the global estimated prevalence of *Cryptosporidium* species has been reported to be 7.6% (Dong et al., 2020).

Transmission of *Cryptosporidium* spp. typically occurs *via* the ingestion of mature oocysts through a faecal-oral route, frequently facilitated by contaminated food or water (Gallas-Lindemann et al., 2016; Ahmed and Karanis, 2018; Ben Ayed et al., 2024), person-to-person, or zoonotic transmission (Sponseller et al., 2014). Following ingestion, oocysts are either expelled in faeces or initiate autoinfection (Chalmers and Davies, 2010). *Cryptosporidium* species exhibit a broad vertebrate host range, with humans and mammals, such as cattle, serving as definitive hosts (Mamedova and Karanis, 2021). Furthermore, many of these species demonstrate distinct host specificities, exemplified by *C. hominis*, primarily infecting humans, *C. tyzzeri*, which targets mice, and *C. cuniculus*, which infects rabbits (Guérin and Striepen, 2020). This is, however, an evolving trait, since the anthroponotic subtypes of *C. parvum* (such as the IIc *gp60* subtypes) have undergone evolutionary adaptation toward increased host specificity, becoming primarily human-associated rather than maintaining the broader infectivity typically observed in subtypes capable of infecting both cattle and a range of other hosts (Guérin and Striepen, 2020).

In immunocompetent individuals, cryptosporidiosis typically presents as a self-limiting diarrhoeal illness (Lee et al., 2016). However, among children, *Cryptosporidium*-associated diarrhoea is a significant cause of mortality, ranking second to rotavirus in children under five years of age (Wang et al., 2020). Cryptosporidiosis exhibits heightened severity in immunocompromised populations, including individuals with HIV/AIDS, organ transplant recipients, and cancer patients (Bouzid et al., 2013; Ahmed et al., 2023). These patients may experience persistent diarrhoea, weight loss, abdominal cramps, fever, nausea, vomiting, and extra-intestinal cryptosporidiosis, affecting the hepatobiliary system, pancreas, upper gastrointestinal tract, and urinary bladder (Bouzid et al., 2013; Dupuy et al., 2021).

Exhibiting substantial genetic diversity, the genus *Cryptosporidium* currently encompasses more than 45 recognized species and over 120 identified genotypes (Mamedova and Karanis, 2021; Ryan et al., 2021a; Tůmová et al., 2023). Classification is based on criteria including morphological, biological, and molecular data (Ryan et al., 2014). Molecular investigations revealed that at least 20 distinct species are implicated in human cryptosporidiosis. However, *C. hominis* and *C. parvum* are the predominant species, accounting for approximately 90% of human infections (Ryan et al., 2014; Thompson and Ash, 2016).

Globally, water-borne cryptosporidiosis is prevalent, and outbreaks originating from contaminated water systems, encompassing sources such as drinking water, recreational water, surface water, groundwater, and wastewater, pose a potential risk of infection to individuals across all age demographics (Karanis et al., 2007). Water-borne pathogens contaminate water sources when faecal matter containing infectious oocysts from infected hosts enters the water system (Mahmoudi et al., 2017). These infections are prevalent in regions with limited access to potable water and adequate sanitation (Ahmed et al., 2018). Across seven regions in Asia and sub-Saharan Africa, *Cryptosporidium* is recognized as the fourth leading etiologic agent of diarrhoea (Checkley et al., 2015). Within the Middle East and North African (MENA) region, *Cryptosporidium* species exhibit an overall prevalence of 24.5%, with Egypt contributing the highest proportion of reported studies (28.5%), followed by Iran (17.1%) (Ben Ayed et al., 2024).

Between 2007 and 2011, *Cryptosporidium* species were identified as the causative agent in 60.3% of reported water-borne outbreaks (Karanis et al., 2007; Baldursson and Karanis, 2011). Subsequently, infection rates increased between 2011 and 2016, with *Cryptosporidium* species accounting for 63% of global protozoan parasite outbreaks (Efstratiou et al., 2017). This proportion rose to 77.4% from 2017 to 2022, with *C. hominis* and *C. parvum* being the most frequently implicated species in these outbreaks (Bourli et al., 2023). Cumulatively, the total number of water-borne outbreaks attributed to *Cryptosporidium* species reached 1227 between the years 2004 and 2023 (Karanis et al., 2007; Baldursson and Karanis, 2011; Efstratiou et al., 2017; Rosado-García et al., 2017; Bourli et al., 2023). Recreational water environments, such as swimming pools, water parks, and hot tubs, represent a significant vehicle of transmission in *Cryptosporidium* outbreaks due to contamination from infected individuals, animals, sewage, or surface runoff (Fewtrell and Kay, 2015; Efstratiou et al., 2017; Bourli et al., 2023).

*Cryptosporidium* spp. exhibit a high degree of adaptation for transmission *via* the faecal-oral route and are implicated in over eight million cases of food-borne illness annually (Ryan et al., 2018). A recent study by Eslahi et al. (2024) reported that the prevalence of *Cryptosporidium* species contamination in fruits and vegetables was highest in Europe (13.32%), followed by the Americas (12.38%), Asia (6.00%), and Africa (4.34%). Pre-harvest *Cryptosporidium*-food contamination can arise from various sources, including soil, faecal matter, irrigation water, insects, domestic and wild fauna, contaminated water employed for pesticide or fungicide application, and human contact. Using compost and manure as fertilizers renders soil a notable source of food contamination, a risk potentially amplified in organic agricultural practices due to increased manure application (Alegbeleye et al., 2018; Golomazou et al., 2024). Post-harvest *Cryptosporidium-*food-contamination can occur due to faecal matter, harvesting implements, transport containers and vehicles, processing machinery, and human handling (Beuchat, 2002).

Globally, 67 documented food-borne outbreaks of cryptosporidiosis have occurred. Notably, *C. parvum* was identified as the causative agent in 96.5% of these cases between 1984 and 2020 (Ahmed and Karanis, 2018; Zahedi and Ryan, 2020). The limited number of reported food-borne outbreaks is attributed to insufficient surveillance systems and the inherent difficulty in tracing the specific food item responsible for the outbreak (Zahedi and Ryan, 2020).

The Asian continent encompasses 58.74% of the global population, exhibiting a population density of 156 individuals per km^2^, with 53.6% of its inhabitants residing in urban centers (Worldometer, 2025). A prior review indicated significant infection rates of *Cryptosporidium* species throughout Asia, demonstrating regional variations (Mahmoudi et al., 2017). The prevalence of *Cryptosporidium* species infection across the Asian continent has been reported to be associated with several identifiable risk factors including water scarcity, deficiencies in waste management infrastructure, the excessive application of manure in agricultural practices, suboptimal hygiene and sanitation standards, the consumption of untreated water, the effects of climate change, and close contact with animals (Bekturganov et al., 2016).

In many Asian countries, environmental conditions and extreme weather phenomena, such as droughts, have played a significant role in contaminating water sources and facilitating the spread of microorganisms (Bekturganov et al., 2016). Extreme weather events, including intense rainfall and flooding, are posited to transport organic fertilizers contaminated with *Cryptosporidium* oocysts into waterways, potentially overwhelming wastewater treatment infrastructure. Conversely, droughts can lead to increased concentration of oocysts in rivers. Also, this season-driven water scarcity situation compels populations to utilize even contaminated water sources (Ahmed et al., 2018). In MENA, a region encompassing Asian countries, *Cryptosporidium* species have been widely reported as prevalent in both surface water and wastewater. These contaminated water sources can serve as reservoirs for oocysts, which may infiltrate soil systems, particularly in areas with poor sanitation or during heavy rainfall and flooding events. Through percolation and subsurface movement, the oocysts-contaminated soil can eventually reach groundwater sources, posing a risk to drinking water supplies if not adequately treated, thereby contributing to human and animal infections (Gallas-Lindemann et al., 2012; Bekturganov et al., 2016; Lim and Nissapatorn, 2017; Alegbeleye et al., 2018; Ben Ayed et al., 2024).

A comprehensive review by Mahmoudi et al. (2017) documented the *Cryptosporidium* epidemiological landscape, genetic diversity, geographical distribution, and transmission dynamics in Asia from 2000 to 2015. Notably, India and China exhibited the highest reported incidence of *Cryptosporidium* infection. The prevalence of infection was found to be heterogeneous, contingent upon the specific population under investigation. *Cryptosporidium* oocysts were detected in surface water throughout Asia, demonstrating frequency and species composition variability. Soil and water were identified as primary vehicles for transmission, with contamination also observed in various vegetables and their wash water. The predominant *Cryptosporidium* species identified across the continent were *C. hominis* and *C. parvum*, alongside less frequent occurrences of *C. felis*, *C. muris*, *C. meleagridis*, and *C. suis* (Mahmoudi et al., 2017).

This review aimed to provide an updated assessment of the *Cryptosporidium* and cryptosporidiosis burden across Asia, extending the temporal analysis by ten years. Specifically, this study focused on the prevalence of *Cryptosporidium* infection within Asian populations and its environmental circulation in water and food sources.

## 2 Materials and methods

### 2.1 Search strategies and data sources

The systematic literature review was conducted in accordance with the PRISMA guidelines (Page et al., 2021) (Supplementary file 1: MeSH keywords and Supplementary Table S1) incorporating defined inclusion and exclusion criteria as well as a bias assessment, as detailed below. To assess the *Cryptosporidium* spp. prevalence and distribution in different countries across the Asian continent, PubMed was used in the literature selection process that started on 6 February 2025 and concluded on 21 March 2025. The articles were searched without language restriction. However, a temporal filter was utilized and applied from 1 January 2015 to 6 February 2025, covering the past 10 years as an update to a previously published review (Mahmoudi et al., 2017).

The search method was confined to title/abstract/keywords utilizing MeSH terms/keywords, with the *Cryptosporidium* and cryptosporidiosis keywords combined with each Asian country name independently, for a total of 50 Asian countries (https://www.countries-ofthe-world.com/countries-of-asia.html) employing Boolean positional operators (AND, OR) (Supplementary file 1: MeSH keywords). The reference list of the included studies was also used to search for related articles for retrieval.

### 2.2 Eligibility criteria and data extraction

Articles with titles indicating the subject of *Cryptosporidium*/cryptosporidiosis in any population were screened and selected as part of the eligibility for inclusion in the literature review. Abstracts and potentially relevant full texts were reviewed independently by six authors (SA, MM, HK, SM, SB, and AN), with any conflicts resolved by consensus (SA, SB, and FM).

For a comprehensive full-text review, the following information was extracted: study site, year of publication, *Cryptosporidium* detection methods, population category, age group for human category, prevalence of *Cryptosporidium*, species/genotypes/subtypes, symptomatology for humans, specifics of symptoms experienced (intestinal and/or extra-intestinal), number of infected, total population sampled, and overall prevalence of *Cryptosporidium* infection, as reported by the authors or estimated from data provided in the paper.

Publications were excluded if they lacked an abstract, a full text, or used languages other than English, or were unrelated to the objective of the study, or had the goal of diagnosis or intervention, or were performed in countries outside of the Asian continent, or were about genetic and/or protein analysis, or were not related to *Cryptosporidium*, or were reviews or case reports or comments and letters to the editors, or if they discussed multicenter or modelling or experimental studies, or if they were publications with confusing data (ambiguous data, poor quality citation, include the same results as another paper published by the same author).

### 2.3 Data analysis

The present review included robust data and multiple variables, such as (i) the number of included studies, (ii) the number of Asian countries, (iii) the number of populations, and (iv) the category of populations.

To simplify data distribution across the Asian continent, Asia was initially divided into five regions: “Central, West, South, East, and Southeast” (World Atlas, 2023). The Asian region was categorized into 50 individual countries according to United Nations Statistics Division (UNSD, 2025), i.e. Afghanistan, Armenia, Azerbaijan, Bahrain, Bangladesh, Bhutan, Brunei, Cambodia, Georgia, China, Cyprus, Iraq, Iran, Indonesia, India, Israel, Japan, Jordan, Kazakhstan, Kuwait, Kyrgyzstan, Laos, Lebanon, Malaysia, Maldives, Mongolia, Myanmar, Nepal, North Korea, Oman, Pakistan, Palestine, Philippines, Qatar, Russia, Saudi Arabia, Singapore, South Korea, Sri Lanka, Syria, Taiwan, Tajikistan, Thailand, Timor-Leste, Turkey, Turkmenistan, United Arab Emirates, Uzbekistan, Vietnam, and Yemen. The included studies were classified into three groups according to the source of *Cryptosporidium*, i.e. humans, water, and/or food.

Due to different population terms used in the analysis, the human populations were categorized into: (i) children (< 1 to < 18 years of age); (ii) adults (18 to > 80 years of age); (iii) both adults and children (BAC) (< 1 to > 80 years of age); (iv) immunocompromised/immunodeficient/immunosuppressed (III) (patients with HIV, cancer/malignancies, on hemodialysis, COVID-19, end-stage renal disease, and malnutrition); (v) individuals posing transmission (IPT) (workers in animal facilities, food handlers, expatriate laborers, farm workers, handlers of domestic animals); and (vi) symptomatic individuals (SI) (diarrhoea patients and those with gastrointestinal symptoms).

Water sources were categorized according to the water type: (i) drinking water (DW) (municipal water and tap water); (ii) surface water (SF) (rivers, floating biofilms, wells, streams, canals, recreational water, irrigation water, pumps, springs, ponds, and lakes); (iii) wastewater (WW) (sewage, slaughter water, and raw/treated wastewater); and (iv) swimming pools (SwP); and (v) mixed water (MW) (treated and untreated water, sewage, and river water).

Food sources are comprised of fruits and vegetables (leafy greens, fresh produce, and raw vegetables. The sources of food comprised wholesale markets (vegetables/fruits sourced from large supermarkets, often involving wholesale distribution, and may be sold in bulk or through various retailers) and retail vendors (specific vendors or sellers directly selling vegetables at riverbanks and farm stalls, or through direct sales to customers).

Mixed sources/categories were the studies that investigated a mixed source of humans and/or water, and/or food. Within each primary source category, subcategories were also identified. Consequently, a single study may involve mixed sources (e.g. humans, water, and/or food) and/or mixed subcategories within a source (e.g. vegetables and fruits under the food category).

A meta-analysis approach was used to determine the significance of *Cryptosporidium* spp. and incidence in different countries of Asia and different categories of populations/samples. Consequently, we extracted data from studies retained by the eligibility criteria section with a subgrouping approach owing to the number and diversity of the target parameters (Ben Ayed et al., 2024). The MedCalc statistical software version 23.2.1 (MedCalc Software Ltd, Ostend, Belgium, https://www.medcalc.org/) was utilized to conduct a proportion meta-analysis to obtain the effect sizes and 95% confidence intervals (95% CI) associated with the variance for the desired set of studies. As measured by Cochran’s score *Q*, the heterogeneity of observations should be observed with *P*_(Q)_ < 0.0005 when tested against a chi-square distribution to conduct correlation analysis. Publication bias was explored in the current analysis and assessed by Egger’s test (*E*), with a low *P*_(E)_-value indicating publication bias.

## 3 Results

### 3.1 Search results

A total of 1838 articles were retrieved from the PubMed database, with 339 duplicates removed. After screening 1499 titles and abstracts, 490 articles were retained for eligibility assessment at the full-text screening stage. Ultimately, 228 original studies were included in the meta-analysis. The selection process and the flowchart of the literature search are presented in (Supplementary file 1: Fig. S1).

### 3.2 Characteristics of the included studies

A total of 228 studies conducted across the Asian continent over the past decade were included and classified across three sources (humans, water, and food). The studies included in this analysis were published between 2015 and the first quarter of 2025, encompassing 56% (28/50) of the countries in Asia. No studies were included from the following 22 countries: Afghanistan, Azerbaijan, Bahrain, Bhutan, Brunei, Georgia, Cyprus, Japan, Kazakhstan, Kuwait, Kyrgyzstan, Laos, Maldives, North Korea, Oman, Palestine, Russia, Sri Lanka, Tajikistan, Timor-Leste, Turkmenistan, and Uzbekistan.

Regarding the human population categories analyzed, children constituted the most studied age group, appearing in 54 studies. Individuals encompassing both adults and children were represented in 42 studies, while adults were exclusively featured in 40 studies. Immunocompromised individuals, individuals posing transmission risks, and symptomatic individuals were reported in 29, 11, and 12 studies, respectively.

The age range of the Asian population examined in the included studies extended from 0 to 99 years. The Asian populations were further categorized based on symptomatic status: symptomatic in 84 studies, asymptomatic in 58 studies, and a combination of both in 9 studies. However, the symptomatic status remained unspecified in 37 studies. Symptomatic individuals exhibited one or more of the following clinical manifestations: diarrhoea, abdominal pain, constipation, flatulence, nausea, vomiting, foul-smelling faeces, and gastrointestinal discomfort. Of particular interest, *Cryptosporidium* was detected in human sputum in a single study (Bagci et al., 2022).

Investigations into water categories revealed that surface water was the most frequently studied, appearing in 23 studies. Wastewater and drinking water were each examined in 8 studies. Swimming pools and mixed water sources were the focus of 3 studies each.

Regarding food sources in Asia, vegetables and fruits were the only food items investigated. Vegetables were the most examined, including raw and leafy greens such as Chinese cabbage, spinach, cauliflower, green onion, and radish, to name a few. Seven studies indicated that vendors (from farms, fields, streets, and riverbanks) were the primary source of these food items.

### 3.3 Features of the prevalence of *Cryptosporidium* spp. in Asia

#### 3.3.1 Overall pooled *Cryptosporidium* prevalence in Asia

Using the total number of explored pooled specimens and the number of pooled infected/contaminated samples investigated in 228 studies, the pooled prevalence of *Cryptosporidium* among the total sample size of 327,783 combining humans, water and food sources, was estimated to be 8.1% (95% CI: 6.9–9.2%; *Q*: 24623.6, *df* = 227, *P*_(Q)_ < 0.0001; *I*^2^: 99.08%, *P*_(E)_ < 0.0001) (Supplementary file 2).

**Fig. 1:**
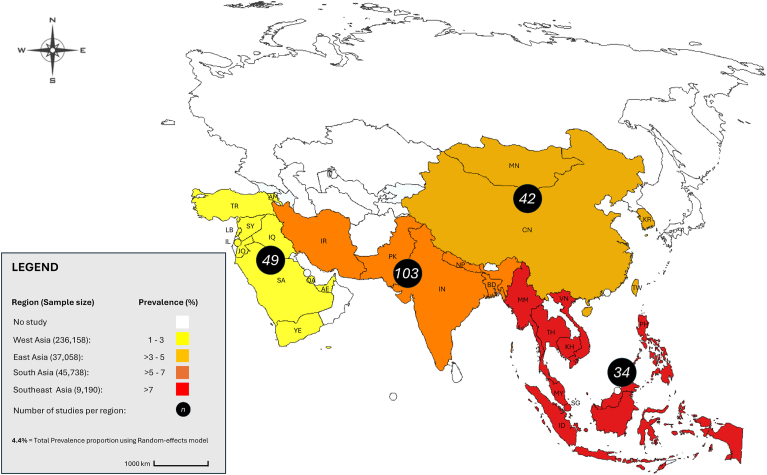
Prevalence of *Cryptosporidium* species in pooled human, water, and food sources by regions in Asia. *Country/Territory codes*: AE, United Arab Emirates; AM, Armenia; BD, Bangladesh; KH, Cambodia; CN, China; IN, India; ID, Indonesia; IR, Iran; IQ, Iraq; IL, Israel; JO, Jordan; KR, Republic of Korea/South Korea; LB, Lebanon; MY, Malaysia; MN, Mongolia; MM, Myanmar; NP, Nepal; PK, Pakistan; PH, Philippines; QA, Qatar; SA, Saudi Arabia; SG, Singapore; SY, Syria; TW, Taiwan; TH, Thailand; TR, Turkey; VN, Vietnam; YE, Yemen (Alpha-2 code ISO 3166 international standard; https://www.iban.com/country-codes). The map template was modified from a free source of https://www.mapchart.net/asia.html.

Four regions (East, South, West, and Southeast Asia) served as the basis for the division in the present review. No eligible paper was retained from Central Asia, with pooled proportions increasing from West Asia (1.3%; 95% CI: 1.3–1.4%); East Asia (3.4%; 95% CI: 3.2–3.6%); South Asia (6.5%; 95% CI: 6.3–6.8%) reaching the highest prevalence within Southeast Asia (7.9%; 95% CI: 7.4–8.5%) (Fig. 1 and Supplementary file 2). The Eastern, Western, Central, Southern, and Southeastern regions accounted for 18.4% (42/228), 21.5% (49/228), 0% (0/228), 45.2% (103/228), and 14.9% (34/228) of the 28 countries with included studies, respectively. The results should be interpreted with caution, as West Asia exhibited the lowest regional prevalence at 1.3% across 49 included studies. Notably, this region contributed the largest sample sizes, with Israel (*n* = 138,909) and Turkey (*n* = 77,945) leading. Additionally, West Asia was home to the highest territorial prevalences across all countries, led by Iraq (58.75%) and Armenia (37.50%).

**Fig. 2:**
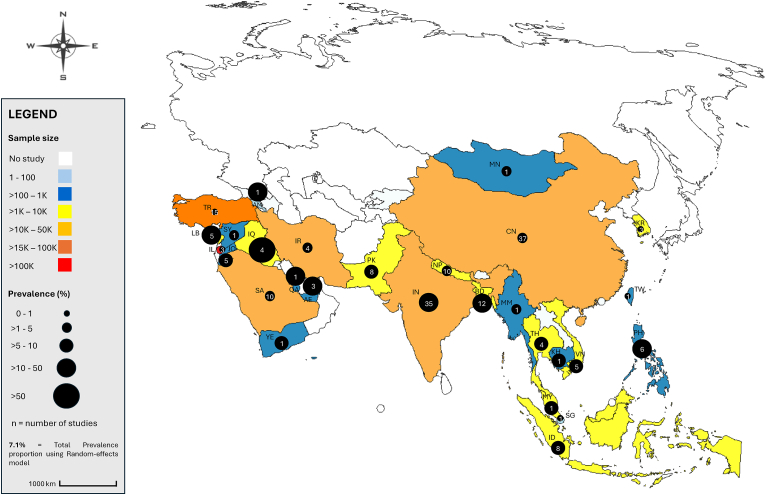
Geographical distribution of *Cryptosporidium* species prevalence in pooled human, water, and food sources in Asia. K = ×1000. *Country/Territory codes*: AE, United Arab Emirates; AM, Armenia; BD, Bangladesh; KH, Cambodia; CN, China; IN, India; ID, Indonesia; IR, Iran; IQ, Iraq; IL, Israel; JO, Jordan; KR, Republic of Korea/South Korea; LB, Lebanon; MY, Malaysia; MN, Mongolia; MM, Myanmar; NP, Nepal; PK, Pakistan; PH, Philippines; QA, Qatar; SA, Saudi Arabia; SG, Singapore; SY, Syria; TW, Taiwan; TH, Thailand; TR, Turkey; VN, Vietnam; YE, Yemen (Alpha-2 code ISO 3166 international standard; https://www.iban.com/country-codes). The map template was modified from a free source of https://www.mapchart.net/asia.html.

Further analysis with country correlation was subsequently explored, since no publication bias was observed according to Egger’s (*P*_(E)_ = 0.136) and Begg’s (*P*_(B)_ = 0.497) tests. Data extracted from the 28 investigated countries (*Q*: 14669.1, *df* = 27, *P*_(Q)_ < 0.0001; *I*^2^: 99.82%) reported pooled *Cryptosporidium* occurrence varying widely from 0% (95% CI: 0–0.1%) within Taiwan to 58.8% (95% CI: 56.7–60.8%) within Iraq (Fig. 2, Supplementary file 1: Fig. S2, and Supplementary file 2). However, the publication bias observed with the country correlation (*P*_(E)_ = 0.001) did not allow for further sub-analysis by this parameter within the different sources (Supplementary file 1: Fig. S3).

There were studies in each of the 28 Asian countries, ranging from one in Armenia, Cambodia, Mongolia, Myanmar, Qatar, Singapore, Syria, Taiwan, and Yemen to as many as 37 in China (Fig. 2).

The aforementioned results from the previous territories should be interpreted with caution, as the actual relationships among countries with the highest and lowest infection rates, sample sizes, and the number of studies reflect a wide range of perspectives. From a prevalence perspective, Iraq reported the highest proportion of 58.8% (four studies), while Taiwan recorded none (0%) (one study), despite both countries having relatively few included studies. Interestingly, sample size patterns revealed further contrasts: Israel had the largest cumulative sample size (138,909 across three studies) yet ranked 26th in prevalence (0.7%), suggesting low infection rates despite extensive sampling. In contrast, Armenia, with just 24 samples from a single study, reported the second-highest prevalence (37.5%). China and India contributed the most studies with 37 and 35 studies, respectively, but did not rank among the highest in prevalence, standing at 18th and 8th, respectively. Several countries, including Armenia, Qatar, and Taiwan, had only one study, yet Armenia and Qatar stood out with notably high prevalences of 37.5% and 15.5%, ranking 2nd and 5th, respectively (Supplementary file 1: Table S2).

An illustration of the overall prevalence of *Cryptosporidium* spp. in Asia over time, based on the year of publication, is presented in Supplementary file 1: Fig. S4. Between 2015 and 2024, no consistent upward or downward trend was observed in the number of studies involving human, water, and food sources collectively. However, pronounced peaks in *Cryptosporidium* detection emerged in 2019 and 2024. Notably, only two studies were retrieved for the first quarter of 2025, suggesting that data for this period remain incomplete (Supplementary file 1: Fig. S4, Supplementary file 2). The previous findings must be interpreted with careful consideration, as the observed increases in *Cryptosporidium* prevalence across different matrices likely result from a complex interplay of multiple contributing factors.

#### 3.3.2 Human *Cryptosporidium* spp. prevalence in Asia

**Fig. 3:**
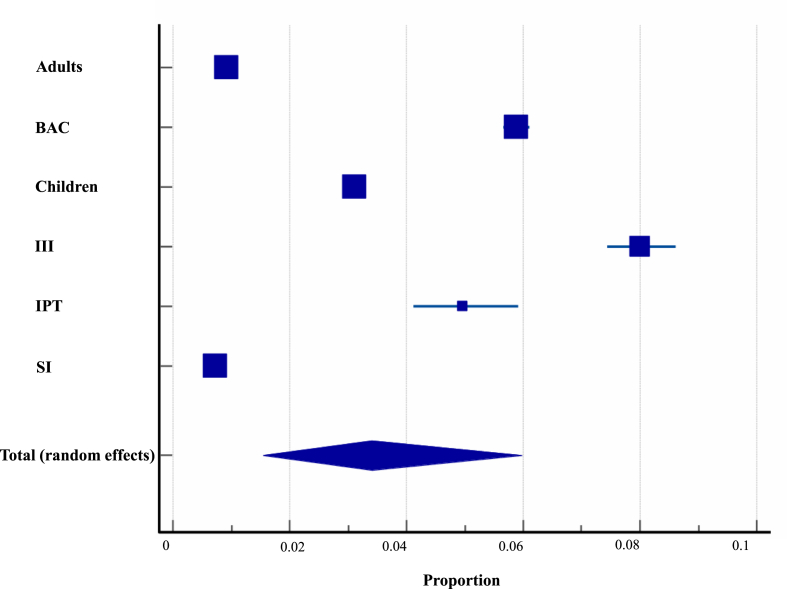
Proportional prevalence of *Cryptosporidium* spp. within the different human populations in Asia. *Abbreviations*: BAC, both adults and children; III, immunocompromised/immunodeficient/immunosuppressed; IPT, individuals posing transmission; SI, symptomatic individuals.

Within the present study, the human source data were extracted from 188 studies covering 27 countries, generating a sample size of 319,821 with an estimated overall prevalence of 5.9% (*Q*: 11812.2, *df* = 26, *P*_(Q)_ < 0.0001; *I*^2^: 99.78%). The current analysis stratified the human population into six categories (see *Section* 2.3). It is worth noting that the lowest prevalence of 0.7% (95% CI: 0.7–0.8%) was observed with the SI category, whereas the highest prevalence of 8.0% (95% CI: 7.4–8.6%) was recorded within the III category (Fig. 3 and Supplementary file 2).

#### 3.3.3 *Cryptosporidium* spp. prevalence in water in Asia

**Fig. 4:**
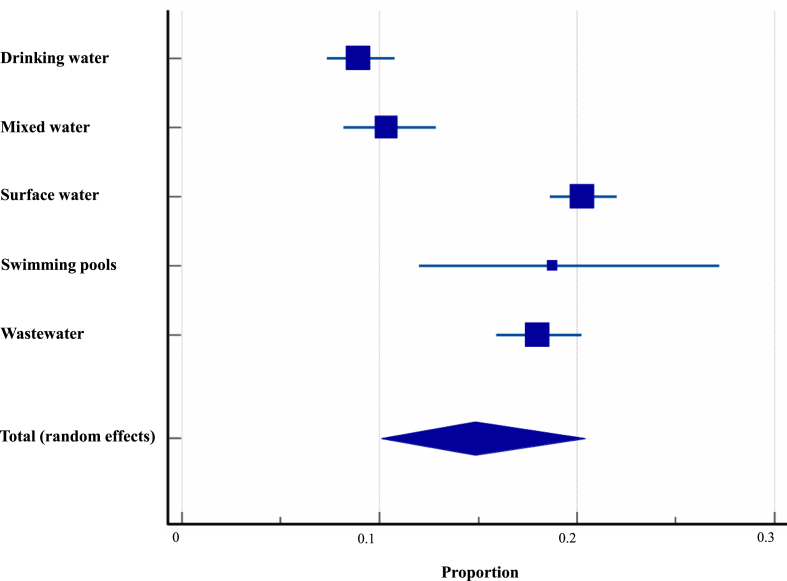
Proportional prevalence of *Cryptosporidium* spp. within different water categories (drinking water, surface water, wastewater, mixed waters and swimming pools) across the Asian continent.

The water data explored within this review was extracted from 44 studies covering 16 countries, generating a sample size of 5448 with an estimated overall prevalence of 23.2%. Since we observed statistically significant heterogeneity indices (*Q*: 1071.1, *df* = 43, *P*_(Q)_ < 0.0001; *I*^2^: 95.99%, *P*_(E)_ = 0.0003), a dependable subgrouping random meta-analysis was conducted for each of the five water categories investigated, i.e. DW, SF, WW, MW, and SwP. The highest *Cryptosporidium* prevalence in Asia was recorded within the surface water category (20.3%; 95% CI: 18.6–21.9%) while the lowest prevalence was observed within the drinking water category (8.9%; 95% CI: 7.3–10.7%) (Fig. 4 and Supplementary file 2).

#### 3.3.4 *Cryptosporidium* spp. prevalence in food in Asia

*Cryptosporidium* contamination in Asian countries was observed in food matrices composed of fruits and fresh produces targeting 2919 samples within ten studies (*Q*: 165.9, *df* = 9, *P*_(Q)_ < 0.0001; *I*^2^: 94.58%) and advancing a random estimated prevalence of 5.6% (95% CI: 2.2–10.4%) without any apparent publication bias (*P*_(E)_ = 0.079, *P*_(B)_ = 0.929). The highest prevalence of 29.6% (95% CI: 17.9–43.6%) was observed by Wei et al. (2019), who reported strawberry farm contamination. When investigating food sources correlation, surprisingly, food from markets had a *Cryptosporidium* prevalence higher than food investigated from vendors (Supplementary file 1: Fig. S5) with 9.3% (95% CI: 5.4–14.7%) and 2.9% (95% CI: 2.4–3.7%), respectively (Supplementary file 2). However, a publication bias was observed within Egger’s test (*P*_(E)_ < 0.0001), possibly due to a significant disparity between the sample sizes of the two categories.

### 3.4 Methods applied and species/subtypes of *Cryptosporidium* in Asia

#### 3.4.1 *Cryptosporidium* spp. in the Asian population

Processing human samples in the Asian studies involved various concentration techniques, including sedimentation and flotation. Certain studies incorporated sputum samples, tissue biopsies, and faecal samples obtained from infected individuals.

For detecting *Cryptosporidium* species, non-molecular methods were employed in 73 out of 188 studies (38.8%), while molecular techniques were utilized in 50 out of 188 studies (26.6%). A combination of both methodologies was implemented in 65 studies (34.6%).

The non-molecular methodologies included wet mount examination with saline and iodine, staining procedures such as modified Ziehl-Neelsen (mZN) and methylene blue staining, rapid immunoassays (RIA), enzyme-linked immunosorbent assays (ELISA), immunofluorescent assays (IFA), immunomagnetic separation followed by IFA (IMS-IFA), and histopathological examination when biopsy samples were included alongside faecal analysis. The molecular methodologies employed included conventional PCR (cPCR), nested PCR (nPCR), restriction fragment length polymorphism PCR (RFLP-PCR), loop-mediated isothermal amplification (LAMP), real-time PCR (qPCR), multiplex PCR, and sequencing. The small subunit ribosomal RNA (SSU rRNA) gene of *Cryptosporidium* spp. was the most frequently targeted locus for molecular identification, followed by the *Cryptosporidium* oocyst wall protein (COWP) gene. While loci like actin and heat-shock protein 70 gene (*Hsp70*) were only occasionally employed for species identification, subtyping efforts predominantly centered around the glycoprotein 60 gene (*gp60*), remained the focal point of most genetic analyses.

Sixty studies were conducted across Asia, focusing on the Asian population (Table 1), and included the genotyping of *Cryptosporidium* isolates. These investigations revealed a variety of *Cryptosporidium* species. In some instances, a single species was identified within a given study, including *C. parvum*, *C. hominis*, *C. meleagridis*, *C. felis*, *C. scrofarum*, and *C. suis*. Conversely, other studies documented multiple *Cryptosporidium* species, while some identified mixed genotypes within individual isolates (Table 1).

**Table 1:** Genotyping/subtyping analysis of *Cryptosporidium* species across the Asian population.

No.	Country (Region)	Study groups (age in years)	GIT symptoms	Molecular methods	Target gene	Species	Subtypes	Reference
1	Bangladesh	Children (<5–14)	–	nPCR, Sequencing	SSU rRNA, *gp60*	*C. hominis*, *C. parvum*	*C. hominis* (IdA15G1, IaA19R3, IbA9G3, IaA23R3)	Karim et al. (2024)
2	Bangladesh (Mymensingh)	Household members	Diarrhoea	nPCR	SSU rRNA, *gp60*	*C. parvum*	IIaA17G2R2	Maxamhud et al. (2025)
3	Bangladesh (Mirpur)	Children (0–2)	Diarrhoea	qPCR	COWP, *gp60*	*C. hominis*, *C. parvum*	N/I	Korpe et al. (2016)
4	Cambodia (Siem Reap)	Children (<16)	Diarrhoea	qPCR, Sequencing	SSU rRNA, *gp60*	*C. canis*, *C. hominis*, *C. meleagridis*, *C. parvum*, *C. suis*, *C. ubiquitum*	*C. hominis* (IaA16R6), *C. parvum* (IIeA7G1)	Moore et al. (2016)
5	China (Wuhan)	Children	Diarrhoea	nPCR, Sequencing	SSU rRNA, *gp60*	*C. meleagridis*	IIIbA21G1R1, IIIbA22G1R1, IIIbA26G1R1	Wang et al. (2017)
6	China (Shanghai)	Mentally disabled children (1 month to 10 years)	Diarrhoea	nPCR, RFLP-PCR	SSU rRNA, COWP	*C. hominis*	IaA14R4	Wang et al. (2018a)
7	China (Zhengzhou, Henan)	Children (1–14)	–	nPCR, Sequencing	SSU rRNA, *gp60*	*C. hominis*, *C. parvum*	*C. parvum* (IIdA19G1)	Yu et al. (2019)
8	China (Binyang)	BAC (5–50)	–	nPCR, Sequencing	SSU rRNA, *gp60*, *Hsp70*	*C. occultus*, *C. viatorum*	*C. viatorum* (XVaA3h)	Xu et al. (2020)
9	China (Jilin)	GIT cancer patients (≤50 to >60)	GIT disturbance	nPCR, Sequencing	SSU rRNA, *gp60*	*C. parvum*	IIaA15G2R1, IIaA15G2R2, IIaA13G2R2	Zhang et al. (2020)
10	China (Qinmu)	Workers in animal facility, villagers	–	nPCR, Sequencing	SSU rRNA	*C. andersoni*, *C. hominis*, *C. parvum*	N/I	Li et al. (2021)
11	China (Southern Xinjiang)	Children (2–6)	–	nPCR, Sequencing	SSU rRNA, *gp60*	*C. felis*, *C. hominis*, *C. parvum*	*C. hominis* (IbA9G3, IdA14, IfA12G1), *C. parvum* (IIdA14G1, IIdA15G1)	Wang et al. (2022)
12	China (Harbin)	HIV (<30 to >50)	–	nPCR, Sequencing	SSU rRNA, *gp60*	*C. cuniculus*, *C. hominis*, *C. meleagridis*	*C*. *meleagridis* (IIIbA23G1R1, IIIeA15G2R, IIIgA26G1R1), *C. hominis* (Ia18R4, IbA20G2)	Zhao et al. (2022)
13	China (Shanghai)	HIV/AIDS patients (18–64)	–	nPCR, Sequencing	SSU rRNA, *gp60*	*C. andersoni*, *C. hominis*, *C. meleagridis*, *C. parvum*	*C. hominis* (IaA28R4, IeA12G3T3)	Jiang et al. (2023)
14	China (Inner Mongolia)	Children (<5)	Diarrhoea	nPCR, Sequencing	SSU rRNA, *gp60*	*C. parvum*	IIdA23G3, IIdA24G3, IIdA24G4, IIdA25G3, IIdA25G4	Guo et al. (2024)
15	China (Heilongjiang)	Villagers	–	nPCR, Sequencing	SSU rRNA, *gp60*	*C. parvum*	IIdA19G1	Hao et al. (2024)
16	China (Wenzhou)	Children	Both diarrhoea and non-diarrhoea	nPCR, Sequencing	SSU rRNA, *gp60*	*C. baileyi*, *C. felis*, *C. parvum*, *C. viatorum*	*C. felis* (XIXa), *C. parvum* (IIdA19G1, IInA10), *C. viatorum* (XVaA3g)	Zhao et al. (2024)
17	India	Transplant recipients and immunocompetent	Diarrhoea	nPCR, Sequencing	SSU rRNA, COWP, DHFR, Cpgp40/15	*C. hominis*, *C. parvum*	*C. hominis* (Ia, Ie, If), *C. parvum* (IId, IIc)	Yadav et al. (2016)
18	India (Assam)	BAC	Diarrhoea	nPCR, RFLP-PCR, Sequencing	SSU rRNA	*C. andersoni*, *C. parvum*	N/I	Hussain et al. (2017)
19	India (Vellore)	Children (3)	Diarrhoea	nPCR, RFLP-PCR	SSU rRNA	*C. felis*, *C. hominis*, *C. parvum*, *C. meleagridis*, Mixed (*C. hominis* + *C. parvum*), Mixed (*C. andersoni* + *C. muris*)	N/I	Kattula et al. (2016)
20	India (Puducherry)	Immunocompromised (11 months to 83 years)	–	cPCR, Sequencing	SSU rRNA	*C. hominis*, *C. parvum*	N/I	Vanathy et al. (2017)
21	India (Delhi)	BAC	Diarrhoea	nPCR, Sequencing	SSU rRNA, *gp60*, CP56, CP47, MSC6-7	*C. hominis*, *C. parvum*	*C. hominis*: CP47 (IA30G16, IA44G28, IA45G29, IA46G30, IA51G35); GP60 (IaA19R3, IbA9G3, IbA10G2, IdA15G1, IdA17G1, IeA11G3T3, IfA14G1)	Yadav et al. (2017)
22	India (West Bengal)	Farm workers	–	qPCR, Sequencing	SSU rRNA, *Hsp70*	*C. parvum*, *C. ryanae*	N/I	Das et al. (2019)
23	India	Haematological malignancy patients	Diarrhoea	nPCR, RFLP-PCR, Sequencing	COWP, SSU rRNA	*C. hominis*	N/I	Ghoshal et al. (2020)
24	India (Kolkata)	BAC (3–50)	Diarrhoea	nPCR, Sequencing	*Hsp70*	*C. hominis*, *C. parvum*, *C. viatorum*	N/I	Sardar et al. (2021)
25	India (Delhi)	Diarrhoea patients (5–36)	Diarrhoea	nPCR, RFLP-PCR, Sequencing	SSU rRNA, *gp60*	*C. felis*, *C. hominis*, *C. parvum*, *C. viatorum*	*C. hominis* (Ia, Ib, Id, Ie), *C. parvum* (IIa, IIc, IId, IIe)	Khalil et al. (2017)
26	Indonesia (Lombok)	BAC	–	cPCR, Sequencing	SSU rRNA	*C. suis*	N/I	Resnhaleksmana et al. (2021)
27	Iran (Tonekabon)	BAC	–	nPCR, Sequencing	SSU rRNA, *gp60*	*C. parvum*	IIaA16G2R1	Shahdoust et al. (2016)
28	Iran (Gonbad Kavoos)	Children	Diarrhoea	nPCR, Sequencing	SSU rRNA, *gp60*	*C. parvum*	IIaA16G2R1, IIaA17G1R1, IIaA22G3R1, IIdA17G1d	Sharbatkhori et al. (2015)
29	Iran (Tabriz)	Children (3 months to 12 years)	Diarrhoea	nPCR, Sequencing	SSU rRNA	*C. parvum*	N/I	Poor et al. (2015)
30	Iran (Fars)	Immunocompetent	GIT disturbance	RFLP-PCR, Sequencing	SSU rRNA	*C. parvum*	N/I	Mohammadpour et al. (2016)
31	Iran (Tehran)	Immunocompetent	GIT disturbance	nPCR, Sequencing	SSU rRNA, *gp60*	*C. parvum*	IIaA16G2R1, IIaA17G1R1, IIdA17G1d	Ranjbar et al. (2016)
32	Iran (Khuzestan)	HIV/AIDS patients	–	PCR, nPCR, RFLP-PCR, Sequencing	SSU rRNA	*C. hominis*, *C. meleagridis*, *C. parvum*	N/I	Ghafari et al. (2018)
33	Iran (Tehran)	Immunodeficient (COVID, cancer, organ transplant)	–	nPCR, Sequencing	SSU rRNA	*C. parvum*	N/I	Esteghamati et al. (2019)
34	Iran (Kurdistan)	Adults	–	nPCR, Sequencing	SSU rRNA, *gp60*	*C. parvum*	IIaA15G2R1	Bahrami et al. (2020)
35	Iran (Central part)	Malignancy (lymphoma, leukemia, organ transplant) (>15)	Both diarrhoea and non-diarrhoea	Semi-nPCR	SSU rRNA	*C. hominis*	N/I	Izadi et al. (2020)
		HIV patients				*C. hominis*, *C. parvum*		
36	Iran (Central part)	Malignancy (lymphoma, leukemia, organ transplant) (>15)	Both diarrhoea and non-diarrhoea	Semi-nPCR	SSU rRNA	*C. hominis*	N/I	Izadi et al. (2020)
37	Iran (Tabriz)	Malnourished children (0–12)	Both diarrhoea and non-diarrhoea	nPCR, LAMP, Sequencing	SSU rRNA	*C. parvum*	N/I	Madadi et al. (2020)
38	Iran (Isfahan)	Cancer patients	–	nPCR, Sequencing	SSU rRNA, *gp60*	*C. hominis*, *C. parvum*	*C. hominis* (IbA6G3), *C. parvum* (IIaA18G3R1, IIaA17G2R1)	Pestechian et al. (2022)
39	Iran (Tehran)	BAC (15 to >50)	–	nPCR, Sequencing	SSU rRNA	*C. parvum*	N/I	Bahadorizadeh et al. (2024)
40	Jordan (Zarqa)	BAC (10 months to 56 years)	Diarrhoea	nPCR, Sequencing	SSU rRNA, *gp60*	*C. hominis*, *C. parvum*	*C. hominis* (IbA9G3, IbA10G2), *C. parvum* (IIdA20G1, IIaA15G2R1)	Hijjawi et al. (2016)
41	Jordan (Al-Mafraq)	Cancer children	Diarrhoea	nPCR, Sequencing	SSU rRNA, *gp60*	*C. parvum*	IIaA17G2R1, IIaA16G2R1	Hijjawi et al. (2017)
42	Jordan (Al-Mafraq, Amman, Irbid, Maan, Zarqa)	BAC (1 month to 54 years)	Diarrhoea	nPCR, Sequencing	SSU rRNA, *gp60*	*C. parvum*	IIaA15G2R1	Hijjawi et al. (2021)
43	Korea (throughout the country)	Children (< 9)	Diarrhoea	nPCR, Sequencing	COWP, SSU rRNA, *gp60*	*C. parvum*	IIaA13G2R1, IIaA14G2R1, IIaA15G2R1	Ma et al. (2019a)
44	Lebanon	Immunocompetent (1–88)	Diarrhoea	nPCR, Sequencing	SSU rRNA, *gp60*	*C. hominis*, *C. parvum*	*C. hominis* (IdA19), *C. parvum* (IIaA15G1R1, IIaA15G2R1)	Osman et al. (2015)
45	Lebanon (Tripoli)	Colon and stomach adenocarcinoma, and immunocompetent	–	qPCR	SSU rRNA	*C. hominis*, *C. parvum*	N/I	Osman et al. (2017)
46	Lebanon (Tripoli)	Schoolchildren (3–16)	Diarrhoea, abdominal pain, vomiting	nPCR, Sequencing	SSU rRNA, *gp60*	*C. hominis*, *C. parvum*	*C. hominis* (IaA18R3, IbA10G2), *C. parvum* (IIaA15G1R1)	Osman et al. (2016)
47	Malaysia (Selangor, Kuala Lumpur, Kelantan)	HIV patients (1–54)	Diarrhoea	nPCR, Sequencing	SSU rRNA	*C. felis*, *C. hominis*, *C. meleagridis*, *C. parvum*	N/I	Iqbal et al. (2015)
48	Myanmar (Shan)	Inhabitants	–	nPCR, Sequencing	SSU rRNA, *gp60*	*C. andersoni*, *C. viatorum*	*C. viatorum* (XVcA2G1c)	Wu et al. (2020)
49	Qatar (Doha)	Children	Diarrhoea	qPCR, RFLP-PCR, Sequencing	SSU rRNA, *gp60*	*C. hominis*, *C. meleagridis*, *C. parvum*	*C. hominis* (IbA9G3, IbA10G2), *C. parvum* (IIdA20G1, IIdA17G1, IIdA18G1, IIdA19G2, IIdA18G2, IIdA16G1, IIdA14G1)	Boughattas et al. (2017)
50	Saudi Arabia (Makkah)	Children (<14)	Diarrhoea	cPCR, RFLP-PCR	SSU rRNA	*C. hominis*, *C. parvum*	N/I	El-Malky et al. (2018)
51	Saudi Arabia (Belgarn)	Food handlers (20–65)	–	qPCR	COWP	*C. parvum*	N/I	Alqarni et al. (2022)
51	Syria (Damascus and Hama provinces)	Children (<5 years)	Diarrhoea	nPCR, RFLP-PCR	SSU rRNA	*C. parvum*	N/I	Kassouha et al. (2016)
53	Thailand (Bangkok)	BAC	–	nPCR, Sequencing	SSU rRNA	*C. parvum*	N/I	Prasertbun et al. (2019)
54	Thailand (Bangkok, Nonthaburi)	Adults (20–65)	Diarrhoea	nPCR, Sequencing	SSU rRNA, *gp60*	*C. canis*, *C. felis*, *C. hominis*, *C. meleagridis*, *C. parvum*, *C. suis*	*C. hominis* (IeA11G3T3, IaA18R3, IaA19R3, IaA20R3, IaA16R3, IfA12G1, IbA9G3, IdA17, IdA11), *C. parvum* (IIoA16G1), *C. meleagridis* (IIIbA19G1R1, IIIbA20G1R1, IIIbA21G1R1b, IIIbA22G1R1c, IIIbA23G1R1b, IIIbA23G1Rc)	Sannella et al. (2019)
55	Thailand (Suan Pheung)	Children (6–11)	–	nPCR, Sequencing	SSU rRNA, *gp60*	*C. felis*	N/I	Sutthikornchai et al. (2021)
56	Turkey (Denizli)	BAC (0–87)	Diarrhoea	nPCR, Sequencing	SSU rRNA	*C. parvum*	N/I	Özkan and İça (2024)
57	Turkey (Van)	Immunosuppressed and immunocompetent (0–65)	Both diarrhoea and non-diarrhoea	nPCR, RFLP-PCR, Sequencing	COWP, *gp60*	*C. parvum*	IIdA15G1, IIdA24G1, IIaA18G3R1, IIdA18G2	Ekici et al. (2022)
58	Turkey (Agri)	Adults (mean age 25.86)	Diarrhoea	nPCR, RFLP-PCR	COWP, *gp60*	*C. parvum*	IIdA18G1, IIdA19G1, IIdA20G1	Aydemir et al. (2024)
59	Vietnam (Nam Dinh)	Diarrhoea patients	Diarrhoea	nPCR, Sequencing	COWP, SSU rRNA, *gp60*	*C. canis*, *C. hominis*	*C. hominis* (IeA12G3T3)	Iwashita et al. (2021)
		Non-diarrhoea individuals	–			*C. canis*	N/I	
60	Vietnam (Bac Giang)	Biogas users	–	nPCR, Sequencing	SSU rRNA	*C. scrofarum*	N/I	Nguyen et al. (2023)

*Abbreviations*: BAC, both adults and children; GIT, gastrointestinal tract; PCR, polymerase chain reaction; RFLP, restriction fragment length polymorphism; *Hsp70*, 70-kDa heat-shock protein gene; cPCR, conventional PCR; nPCR, nested PCR; qPCR, real-time PCR; LAMP, loop-mediated isothermal amplification; COWP, *Cryptosporidium* oocyst wall protein; *gp60*, 60-kDa glycoprotein gene; SSU-rRNA, small subunit ribosomal RNA; HIV, human immunodeficiency virus; N/I, not investigated.

#### 3.4.2 *Cryptosporidium* spp. in Asian water resources

Non-molecular methods to detect *Cryptosporidium* sp. in water resources were used in 26.7% (12/45) of the studies, while molecular techniques were used in 44.4% (20/45). Thirteen studies (28.8%) combined both methods.

The Asian studies meticulously prepared water samples using various concentration techniques (sedimentation and filtration). The non-molecular methods employed wet mount examination (saline and iodine), mZN, ELISA, IFA, IMS-IFA, and scanning electron microscopy (SEM). The molecular methods employed in the analyzed studies comprised cPCR, nPCR, RFLP-PCR, and sequencing. The *Cryptosporidium* SSU rRNA was the most frequently targeted locus for molecular identification. Except for a single study that utilized the *Hsp70* locus (Ehsan et al., 2015) for genotyping/subtyping, *gp60* was the most targeted locus in the included studies.

In 20 genotyping studies focusing on water sources, a variety of *Cryptosporidium* species were identified, with four of these studies further extending their investigation to subtyping (Table 2). Certain species were exclusive within individual studies, including *C. parvum*, *C. hominis*, *C. ryanae*, and *C. suis*. Conversely, other studies documented multiple *Cryptosporidium* species, while some revealed the existence of mixed genotypes within single isolates.

**Table 2:** Genotyping/subtyping analysis of *Cryptosporidium* species within Asian water resources.

No.	Country (City)	Type of water investigated	Sampling volume (L)	Molecular method	Target gene	Species	Subtype	Reference
1	Bangladesh (Mymensingh)	SF (ponds)	15	nPCR	SSU rRNA, *Hsp70*	*C. andersoni*, *C. hominis*	N/I	Ehsan et al. (2015)
2	China (Shanghai)	WW (treatment plant)	30	nPCR, Sequencing	SSU rRNA, *gp60*	*C. baileyi*, *C. canis*, *C. felis*, *C. hominis*, *C. meleagridis*, *C. muris*, *C. parvum*, *Cryptosporidium* rat genotype I, *Cryptosporidium* rat genotype IV, *C. suis*-like	*C. hominis* and *C. parvum* (N/D), *C. meleagridis* (IIIbA22G1R1c)	Ma et al. (2016)
3	China (Tianjin)	SF (lakes)	20	nPCR, Sequencing	SSU rRNA	*C. andersoni*, *C. fragile*, *C. meleagridis*, *C. parvum*, *C. ubiquitum*	N/I	Xiao et al. (2016)
4	China (Shanghai)	WW (combined sewer overflow)	3	nPCR, Sequencing	SSU rRNA, *gp60*	*C. baileyi*, *C. hominis*, *C. meleagridis*, *C. muris*, *C. parvum*, *C. ubiquitum*, *C. viatorum*	*C. hominis* (IaA18R4, IbA19G2), *C. parvum* (IIdA19G1)	Huang et al. (2017)
		WW				*C. baileyi*, *C. felis*, *C. hominis*, *C. meleagridis*, *C. muris*, *C. parvum*, *Cryptosporidium* rat genotype I, *Cryptosporidium* rat genotype IV, *C. ubiquitum*, *C. viatorum*	*C. hominis* (IaA18R4, IbA19G2), *C. parvum* (IIdA19G1), *C. ubiquitum* (XIIg, XIIh), *C. meleagridis* (IIIbA22G1R1c), *C. viatorum* (XVaA6a)	
5	China (Beijing)	SwP	10	nPCR	SSU rRNA	*C. hominis*, *C. parvum*	N/I	Xiao et al. (2017)
6	China (Tianjin)	SF (recreational lakes)	20	nPCR	SSU rRNA	*C. andersoni*, *C. fragile*, *C. hominis*, *C. meleagridis*, *C. parvum*, *C. ubiquitum*	N/I	Xiao et al. (2018)
7	China (Qinghai Tibetan Plateau Area)	SF (river)	20	nPCR, sequencing	SSU rRNA	*C. andersoni*, *C. hominis*	N/I	Ma et al. (2019b)
		MW (sewage and rivers)	20	nPCR, Sequencing	SSU rRNA	*C. andersoni*, *C. canis*, *C. hominis*, *C. parvum*, *C. struthionis*	N/I	
8	China (Shanghai)	WW	0.5–1.0	nPCR, RFLP PCR, Sequencing	SSU rRNA, *gp60*	*C. hominis*, *C. meleagridis*, *C. parvum*, Mixed (*C. hominis* + *C. meleagridis*), Mixed (*C. hominis* + *C. meleagridis* + *C. parvum*)	*C. hominis* (IaA13R4, IaA14R4, IbA19G2, IdA19), *C. meleagridis* (IIIbA18G1R1, IIIbA21G1R1da, IIIbA24G1R1c)	Jiang et al. (2020)
9	China (Guangzhou)	WW (raw)	1	nPCR, Sequencing	SSU rRNA, *gp60*	*C. baileyi*, *C. felis*, *C. parvum*, *Cryptosporidium* rat genotype IV	*C. parvum* (IIdA15G1)	Fan et al. (2021)
		WW (treatment plants)				*C. baileyi*, *C. bovis*, *C. canis*, *C. felis*, *C. meleagridis*, *C. muris*, *C. occultus*, *C. parvum*, *Cryptosporidium* rat genotype I, *Cryptosporidium* rat genotype IV, *C. serpentis*	*C. parvum* (IIdA15G1)	
10	China (Guiyang)	SF (lakes)	0.5	nPCR, Sequencing	SSU rRNA, *gp60*	*C. hominis*, *C. parvum*	*C. hominis* (IaA13R8, IeA11G3T3), *C. parvum* (IIpA9)	Jia et al. (2022)
11	China (Shanghai)	WW	0.6–0.8	nPCR, Sequencing	SSU rRNA, *gp60*	*C. hominis*	*C. hominis* (IdA14)	Jiang et al. (2024)
12	India (Odisha)	SF	20	nPCR	SSU rRNA	*C. hominis*	N/I	Daniels et al. (2015)
		GW (deep)						
		GW (shallow)						
		DW						
13	India (West Bengal)	WW (farm water)	1–2	nPCR, Sequencing	SSU rRNA, *Hsp70*	*C. ryanae*	N/I	Das et al. (2019)
14	Iran (Tehran)	SF (river)	50	nPCR, Sequencing	SSU rRNA, *gp60*	*C. andersoni*, *C. canis*, *C. hominis*, *C. muris*, *C. parvum*	*C. parvum* (IId), *C. hominis* (Id)	Mahmoudi et al. (2015)
15	Iran (Tehran)	WW (raw)	5	nPCR, Sequencing	SSU rRNA	*C. andersoni*, *C. xiaoi*	N/I	Hatam-Nahavandi et al. (2016)
16	Korea (Seoul, Gimhae, Daejeon)	SF (lake, river, water intake plants)	40	nPCR	SSU rRNA	*C. parvum*	N/I	Bahk et al. (2018)
17	Pakistan (Faisalabad)	WW (sewage water)	5	nPCR	SSU rRNA	*C. parvum*	N/I	Abbas et al. (2022)
		DW (municipal water)						
		SF (canal)						
18	Philippines, Thailand, and Malaysia	MW (treated and untreated water)	0.015 sediment^a^	qPCR	N/M	*C. parvum*	N/I	Kumar et al. (2016)
19	Philippines (Rizal, Laguna, Metro Manila)	SF (lake basin)	1	nPCR, Sequencing	SSU rRNA	*C. baileyi*, *C. galli*, *C. hominis*, *C. muris*, *C. parvum*, *Cryptosporidium* rat genotype IV, *C. suis*	N/I	Dela Peña et al. (2021)
20	Vietnam (Hanam)	SF (river)	5	nPCR, Sequencing	SSU rRNA	*C. suis*	N/I	Nguyen et al. (2016)
		WW (sewage)	2					
		SF (pond)	5					
		SF (canal)	2					

*Abbreviations*: DW, drinking water; GW, ground water; MW, mixed water; SwP, swimming pools; SF, surface water; WW, wastewater; PCR, polymerase chain reaction; nPCR, nested PCR; RFLP-PCR, restriction fragment length polymorphism-PCR; qPCR, real-time PCR; SSU rRNA, small subunit ribosomal ribonucleic acid; *Hsp70*, heat-shock protein 70 gene; *gp60*, 60-kDa glycoprotein gene; N/I, not investigated; N/M, not mentioned; N/D, not detected (An investigation was conducted; however, it did not succeed in identifying any subtypes).

a The study did not specify the sample volume but noted that processing followed the U.S. EPA Methods 1622/1623.1.

The volume of water analyzed in the Asian studies ranged from 15ml to 50l (Table 2). The mean volumes of water examined varied across the different water categories as follows: DW (50ml to 20l), MW (20–161l), SF (500ml to 50l), SwP (8–50l), WW (500ml to 30l). The concentration of *Cryptosporidium* oocysts/l of water was assessed in 16 studies, with reported values ranging from 0 to 80,000 oocysts/l (Table 3). Notably, the highest oocyst concentration (80,000 oocysts/l) was reported in the Philippines in a mixed water source comprising tap water, spring water, and groundwater. Surface water constituted the second category with a high oocyst concentration in Nepal (126–794 oocysts/l).

**Table 3:** The concentrations of *Cryptosporidium* oocysts per liter in Asian water resources.

No.	Country (City)	Type of water	Oocyst concentration^a^	Reference
1	Armenia	SF (river)	0.4–2.0	Shcherbakov et al. (2024)
2	Bangladesh (Mymensingh)	SF (ponds)	0.02–1.1	Ehsan et al. (2015)
3	China (Shanghai)	WW (treatment plants)	0–0.93	Ma et al. (2016)
4	China (Tianjin)	SF (lake)	3.65	Xiao et al. (2016)
5	China (Xuzhou)	SF (lake)	0–0.8	Kong et al. (2017)
6	China (Beijing)	SwP	0.3	Xiao et al. (2017)
7	China (Guangzhou)	SwP	0.03–0.14	Wei et al. (2018)
8	China (Tianjin)	SF (recreational lake)	3.6	Xiao et al. (2018)
9	China (Guangzhou)	SF (irrigation water)	0.1–0.2	Wei et al. (2019)
10	Korea (Seoul, Gimhae, Daejeon)	SF (lake, river, water intake plants)	0–36.0	Bahk et al. (2018)
11	Lebanon (Shatila Refugee Camp in Beirut)	DW (public and private wells, and municipal tap)	0–5.0	Khoury et al. (2016)
12	Malaysia (Sarawak)	DW	0.02–0.06	Richard et al. (2016)
13	Nepal	SF (river)	126–794	Tandukar et al. (2018)
14	Philippines (Manila)	SF (pump)	0.1	Labana et al. (2018)
		SF (river)	0.4	
		SF (creek)	0.8	
15	Philippines (Laguna)	MW (tap water, spring water, and groundwater)	80,000	Paller et al. (2022)
16	Philippines	MW (treated and untreated water)	0.06	Kumar et al. (2016)
	Malaysia		0.57	
	Thailand,		0.22	

*Abbreviations*: DW, drinking water; MW, mixed water; SwP, swimming pools; SF, surface water; WW, wastewater.

a Number of oocysts per liter.

#### 3.4.3 *Cryptosporidium* spp. in Asian food resources

For detecting *Cryptosporidium* spp. in food resources, non-molecular methods were employed in 30% (3/10) of the studies, whereas molecular techniques were utilized in 50% (5/10). Two studies (20%) integrated non-molecular and molecular approaches.

The Asian studies under review employed meticulous methodologies for preparing food samples to detect *Cryptosporidium* oocysts. These techniques included washing, elution, and purification of the food matrices. Subsequently, purified oocysts underwent concentration *via* sedimentation and flotation procedures. The non-molecular methods employed were IFA and IMS-IFA. The *Cryptosporidium* SSU rRNA, COWP, and *gp60* genes were the loci targeted for molecular identification.

In the six studies focusing on *Cryptosporidium* species in food (Table 4), *C. parvum*, *C. andersoni*, and *C. suis* were the species reported. Subtyping analysis was not successful for any of the *Cryptosporidium* species investigated in the included studies in food resources.

**Table 4:** Molecular analysis of *Cryptosporidium* species within Asian food resources.

No.	Country (City)	Type of food investigated (specific material)	Sample source	Molecular method	Target gene	Species^a^	Reference
1	China (Henan)	Vegetables (Chinese chive)	Farms and markets	nPCR, Sequencing	SSU rRNA, *gp60*	*C. parvum*	Li et al. (2019)
2	China (Qinghai)	Vegetables (Puha)	Vendors	nPCR	SSU rRNA	*C. andersoni*	Li et al. (2020)
		Vegetables (Chinese cabbage, Chinese chives, wild cabbage, lettuce, asparagus lettuce leaves, maize cobs peels, Romaine lettuce, amaranth)				*C. parvum*	
3	India (Chandigarh)	Vegetables (fresh produce)	Vendors and markets	nPCR	SSU rRNA, COWP	*C. parvum*	Utaaker et al. (2017)
4	Korea (Seoul)	Vegetables (*Perilla* leaves, winter-grown cabbages, chives, sprouts, blueberries, cherry tomatoes)	Fields	qPCR, Sequencing	Rad 16 ortholog gene	*C. parvum*	Sim et al. (2017)
5	Pakistan (Faisalabad)	Vegetables (raw)	Vendors and markets	nPCR, Sequencing	SSU rRNA	*C. parvum*	Abbas et al. (2022)
6	Vietnam (Hanam)	Vegetables (leafy greens)	Riverbanks	nPCR, Sequencing	SSU rRNA	*C. suis*	Nguyen et al. (2016)

*Abbreviations*: PCR, polymerase chain reaction; nPCR, nested PCR; qPCR, real-time PCR; SSU rRNA, small subunit ribosomal ribonucleic acid; COWP, *Cryptosporidium* oocyst wall protein; *gp60*, 60-kDa glycoprotein gene.

a Subtyping analysis was not successful for any of the *Cryptosporidium* species investigated in the studies included.

### 3.5 Frequencies of *Cryptosporidium* species in Asia

Twenty-three distinct species and four instances of mixed species infections were presented in different sources from humans, water, and food (Table 5). Mixed *C. hominis* + *C. parvum* and *C. andersoni* + *C. muris* infections were identified in the Asian population, and mixed *C. hominis* + *C. meleagridis* and *C. hominis* + *C. parvum* + *C. meleagridis* were identified within the Asian water sources (Table 5). Notably, *C. parvum*, *C. hominis*, and *C. meleagridis* were the common *Cryptosporidium* species identified across human and water sources, whereas *C. parvum* was the common species in food sources. *Cryptosporidium parvum* exhibited the highest prevalence across all three sources.

**Table 5:** Frequencies of *Cryptosporidium* species in Asia across human populations, water sources, and food resources. Numbers in bold indicate the most frequent species per source.

No.	Species	No. of documented species per source			Total frequency
		Humans	Water	Food	
1	*C. andersoni*	5	7	1	13
2	*C. baileyi*	1	6	–	7
3	*C. bovis*	–	1	–	1
4	*C. canis*	4	4	–	8
5	*C. suis*	3	3	1	7
6	*C. felis*	7	4	–	11
7	*C. serpentis*	–	1	–	1
8	*C. muris*	–	6	–	6
9	*C. meleagridis*	8	7	–	15
10	*C. parvum*	**51**	**16**	**5**	**72**
11	*C. hominis*	**32**	**14**	–	**46**
12	*C. fragile*	–	2	–	2
13	*C. ubiquitum*	1	4	–	5
14	*Cryptosporidium* rat genotype IV	–	4	–	4
15	*C. occultus*	1	1	–	2
16	*C. galli*	–	1	–	1
17	*Cryptosporidium* rat genotype I	–	3	–	3
18	*C. xiaoi*	–	1	–	1
19	*C. ryanae*	1	1	–	2
20	*C. struthionis*	–	1	–	1
21	*C. viatorum*	5	1	–	6
22	*C. culuculus*	1	–	–	1
23	*C. scrofarum*	1	–	–	1
1-M	*C. hominis* + *C. meleagridis*	–	1	–	1
2-M	*C. hominis* + *C. meleagridis* + *C. parvum*	–	1	–	1
3-M	*C. hominis* + *C. parvum*	1	–	–	1
4-M	*C. andersoni + C. muris*	1	–	–	1
	Total number of species per source	14	21	3	

*Abbreviation*: M, mixed species.

The Asian human population, was found to be infected with 14 different *Cryptosporidium* species (Table 5). Various Asian water sources were found to be contaminated with 21 different *Cryptosporidium* species. Based on the frequency of their documentation across the included studies, the most common species, presented in descending order, were *C. parvum*, *C. hominis*, followed by equal presentation of *C. meleagridis* and *C. andersoni* (Table 5). Different Asian food resources were contaminated with three *Cryptosporidium* species (Table 5), with *C. parvum* being the most frequently identified species (Table 5).

### 3.6 Frequencies of *Cryptosporidium* subtypes in Asia

Subtyping analyses were conducted within the Asian population and water resources. Six *Cryptosporidium* species and their reported *gp60* subtypes families were examined (Table 6): *C. parvum* (6 subtypes), *C. hominis* (5 subtypes), *C. meleagridis* (3 subtypes), *C. ubiquitum* (2 subtypes), *C. felis* (1 subtype), and *C. viatorum* (1 subtype).

**Table 6:** Frequencies of *Cryptosporidium gp60* subtype families in Asia across human populations and water sources. Numbers in bold indicate the most frequent subtype per source.

Species	Subtype family	No. of documented subtype families per source		Total frequency
		Humans	Water	
*C. hominis*	Ia	**18**	**4**	22
	Ib	**11**	3	14
	Id	6	3	9
	Ie	5	1	6
	If	3	–	3
*C. parvum*	IIa	**28**	–	28
	IIc	2	–	2
	IId	**27**	**5**	32
	IIe	2	–	2
	IIo	1	–	1
	IIp	–	1	1
*C. meleagridis*	IIIb	9	**3**	12
	IIIe	1	–	1
	IIIg	1	–	1
*C. ubiquitum*	XIIg	–	1	1
	XIIh	–	1	1
*C. felis*	XI	1	–	1
*C. viatorum*	XV	3	1	4

Within the Asian population, 15 *Cryptosporidium gp60* subtype families were identified. The most prevalent *Cryptosporidium* subtype families were *C. parvum* IIa and *C. parvum* IId, with a near-equal reporting frequency of 28 and 27 studies, respectively, followed by *C. hominis* subtypes Ia and Ib (Table 6).

Various Asian water resources were found to be contaminated with subtypes of nine distinct *Cryptosporidium* families. Among these, *C. parvum* IId, *C. hominis* Ia, and *C. meleagridis* IIIb were the most frequently encountered subtypes.

It is noteworthy that the following *Cryptosporidium* subtype families were shared between human populations and water resources in Asia, presented in descending order of frequency: *C. parvum* IId, *C. hominis* Ia, *C. meleagridis* IIIb, and *C. parvum* Ib, Id, and Ie (Table 6).

## 4 Discussion

The present review offers insights into the prevalence and distribution of *Cryptosporidium* species in human populations, water sources, and food resources across the Asian continent in the past decade.

Our analysis indicated the overall prevalence of *Cryptosporidium* spp. in Asia to be 8.1% between 2015 and 2025, aligning with a systematic review and meta-analysis encompassing the global population, in which the estimated pooled prevalence of *Cryptosporidium* infection was 7.6% (Dong et al., 2020). The overall *Cryptosporidium* prevalence per Asian region demonstrated the highest prevalence proportion in the Southeast Asia and the absence of Central Asia representation. However, such an interpretation should consider the number of studies and the sample size associated with each region. The Southeast Asia region is acknowledged as an “epicenter” for emerging infectious diseases. The tropical and subtropical climate of Southeast Asia, characterized by high humidity and frequent rainfall, creates favorable conditions for the survival and transmission of *Cryptosporidium* oocysts in the environment. In many parts of the region, rapid urbanization and population growth induced inadequate sanitation infrastructure and limited access to clean water, facilitating faecal-oral transmission, particularly in densely populated or low-resource settings. Furthermore, the region’s diagnostic limitations, noting that many countries of Southeast Asia lack access to sensitive molecular tools, leading to underreporting and insufficient understanding of species distribution and transmission dynamics (Lim and Vythilingam, 2013, Lim et al., 2013; Bordier and Roger, 2013; Hashim and Hashim, 2016; Lan et al., 2016; Utami et al., 2020).

The considerable variation in *Cryptosporidium* prevalence across Asian countries, from 0% in Taiwan to 58% in Iraq, is largely influenced by environmental and infrastructural factors, such as poor sanitation, limited access to clean water, inadequate sewage systems, and population displacement from political conflicts. Additionally, differences in diagnostic methods, where molecular techniques detect more cases than traditional tests, along with variations in study populations, public health surveillance, sample size, study design, and sociocultural practices, might also impact prevalence estimates in the wide area of the Asian continent.

The available data in the present study might be affected by a geographical bias, as a disproportionate number of studies have been conducted in certain countries, notably Taiwan (one study) and China (37 studies), while limited research is available from other regions, particularly Central Asia. This under- or over-representation may skew the pooled prevalence estimates and limit the generalizability of the results to the broader region. Such an imbalance highlights the need for more epidemiological studies in underexplored regions to obtain a more accurate and comprehensive understanding of *Cryptosporidium* spp. prevalence across Asia.

Significant surges in *Cryptosporidium* spp. detection observed in 2019 and 2024 cannot be attributed to an increase in the overall number of studies. Rather, these spikes stemmed from a higher proportion of research focused on human subjects, accounting for 79.1% (19/24) of studies in 2019 and 87% (20/23) in 2024. The volume of research conducted before the COVID-19 pandemic (2015–2019) and after it (2020–2025) appears relatively consistent. However, it is important to highlight that the prevalence recorded in 2024 surpassed that of 2019. The observed rise in human cases may be attributed to the broader application of molecular diagnostic techniques, as well as an increase in the number of parasitological stool examinations that include the detection of *Cryptosporidium* spp. In Europe, the growing number of reported cases is largely associated with enhanced detection efforts, rather than an actual increase in prevalence, which appears to remain stable. This trend reinforces the urgency of expanding research on this water-borne protozoan parasite.

The pooled prevalence of *Cryptosporidium* spp. in the Asian population stands at a modest 5.9%, significantly trailing behind Africa’s 21–50% and the Americas’ 9–21% (Jann et al., 2022; Omolabi et al., 2022; Ahmed et al., 2023). This lower rate is likely reflected by the data collected over just the past decade, highlighting regional differences in infection patterns across continents.

This review underscores that *Cryptosporidium* spp. infection predominantly affects individuals with compromised immune systems (8.0%), such as those living with HIV, cancer patients, organ transplant recipients, and individuals affected by COVID-19, corroborating findings from earlier research in Asia (Mahmoudi et al., 2017). Worldwide estimates reveal a prevalence ranging from 11% to 14% among HIV-positive populations, with rates reaching up to 25% in certain African regions, largely influenced by hygiene-related factors (Wang et al., 2018b; Ahmadpour et al., 2020; Omolabi et al., 2022). Among organ transplant recipients, infection rates are approximately 15% (Ahmed et al., 2024). The immune competence of the host plays a pivotal role in determining the severity and prognosis of cryptosporidiosis, which can be severe, prolonged, and occasionally fatal in these vulnerable groups (Ryan et al., 2016; Semmani et al., 2023).

*Cryptosporidium* oocysts have been detected at high levels in surface water across Asia in the last decade, with an overall prevalence of 20.3%, aligning with the 45.3% global prevalence of *Cryptosporidium* spp. based on water type (Daraei et al., 2021). This widespread presence highlights growing concerns over surface water quality and safety, given its vulnerability to environmental contamination and its crucial role in supporting human and ecological needs. Surface waters are particularly susceptible to contamination from sewage discharges and agricultural runoffs, where livestock proximity to water sources, intensive farming practices, and manure application on fields led to the high rates of water-borne cryptosporidiosis (Bourli et al., 2023).

It is alarming that *Cryptosporidium* contamination in Asian drinking water reaches a prevalence of 8.9%; such a level indicates the potential for widespread and severe water-borne transmission, posing a critical public health threat. *Cryptosporidium* spp. are resilient parasites that can cause severe diarrheal illness, especially in vulnerable populations like children, the elderly, and immunocompromised individuals. Contaminated drinking water serves as a primary infection source, leading to outbreaks and sustained transmission. *Cryptosporidium* spp. has been identified as the leading cause of 1227 reported water-borne outbreaks (Karanis et al., 2007; Efstratiou et al., 2017; Rosado-García et al., 2017; Bourli et al., 2023). This high contamination rate reflects inadequate water treatment and sanitation infrastructure, increasing the risk of large-scale infections and associated morbidity and mortality across communities. Immediate interventions are therefore essential to ensure safe drinking water and prevent further health crises.

*Cryptosporidium* contamination in Asian countries has been detected in various food matrices, including fruits and fresh produce, with a prevalence reaching 5.6%. *Cryptosporidium* is recognized as one of the top ten food-borne pathogens by the FoodNet surveillance programme in the USA (Crim et al., 2014; Ali et al., 2024), and cryptosporidiosis is recognised as the second most significant food-borne illness in western and northern Europe (Robertson et al., 2015; Bouwknegt et al., 2018; Ali et al., 2024). Globally, there have been 67 reported food-borne outbreaks of cryptosporidiosis (Ahmed and Karanis, 2018; Zahedi and Ryan, 2020). Contamination often occurs during food processing, with oocysts adhering to the moist surfaces of fruits and vegetables, posing ongoing risks to food safety (Ryan et al., 2018; Ali et al., 2024).

Surprisingly, food samples from markets exhibited a significantly higher *Cryptosporidium* prevalence of 9.3% compared to 3.0% in vendor-sourced foods. However, Egger’s test revealed a publication bias, likely influenced by the large disparity in sample sizes between these groups. The increased contamination in markets may stem from more complex handling, extended storage, and transportation processes, which elevate the risk of food contamination with oocysts. Markets also consolidate produce from diverse origins, heightening cross-contamination potential, particularly where hygiene standards are inconsistent. Conversely, vendors typically manage smaller quantities with shorter supply chains, possibly reducing contamination risks. Additionally, market environments, especially in densely populated or poorly regulated areas, may lack rigorous sanitation, further contributing to the higher prevalence observed.

In the present review, non-molecular methods were more commonly employed than molecular techniques for detecting *Cryptosporidium* spp. in Asian studies, in agreement with microscopy being the predominant approach in South and Central America, while equal use of microscopy and molecular diagnostics was shown for North America (Jann et al., 2022).

A clear predominance of *C. parvum* (subtype families IIa and IId) and *C. hominis* (subtype families Ia and Ib) was observed across the Asian population and regional water sources, while *C. parvum* appears more frequently in food. This pattern aligns with global trends, where *C. parvum* and *C. hominis* remain the most prevalent species infecting humans (Feng et al., 2018; Ryan et al., 2021b), and mirrors previous findings from Asia (Mahmoudi et al., 2017). In the MENA region, *C. parvum* and *C. hominis* were consistently identified across nearly all studies (Ben Ayed et al., 2024). *Cryptosporidium hominis* is the predominant species responsible for water-borne cryptosporidiosis outbreaks worldwide (Bourli et al., 2023), whereas food-borne outbreaks are more commonly linked to *C. parvum*, reflecting its strong zoonotic transmission potential (Zahedi et al., 2020; Ali et al., 2024). This distinction highlights the differing epidemiological dynamics of each transmission route.

The concurrent presence of *C. parvum* and *C. hominis* in the Asian population, as well as in food and water sources, reveals a complex interplay between anthroponotic and zoonotic transmission pathways. *Cryptosporidium hominis* predominance highlights human-to-human transmission and points to sewage-related contamination, especially in water-borne outbreaks (Robertson et al., 2020), while *C*. *parvum* frequently linked to livestock signals environmental contamination through agricultural practices and inadequate sanitation. The detection of *C. parvum* in both food and water emphasizes the fragility of public health infrastructure in densely populated or resource-limited regions, presenting a substantial risk for widespread outbreaks. Supporting this concern, out of 40 reported cryptosporidiosis outbreaks in the past decade, 27 were linked to water and only 13 to food contamination, reinforcing the greater vulnerability of water systems (Zahedi and Ryan, 2020; Ali et al., 2024). This disparity is likely due to the under-ascertainment of food-borne outbreaks, which are considerably more difficult to identify and monitor. Water sources generally serve a defined population, making outbreak detection and tracing more straightforward. In contrast, contaminated food products often have variable distribution, and only certain individuals, depending on their dietary habits, may be exposed. Moreover, fresh produce is typically no longer available by the time clinical cases are recognized, and investigations begin. In comparison, water may still be accessible for testing or, if contamination is persistent, continue to pose an ongoing threat.

The detection of an exceptionally high concentration of *Cryptosporidium* oocysts, 80,000 oocysts/l in a mixed water source in the Philippines, alongside elevated levels in Nepalese surface water (126–794 oocysts/l) (Paller et al., 2022) underscores a critical public health concern. One of the studies conducted in the Philippines (Masangkay et al., 2020) has similarly reported a high oocyst density using a comparable low-volume approach (50ml), thereby reinforcing the validity of the findings. While the low-volume approach may raise methodological concerns, it was justified by contextual relevance, specifically, the realistic risk of human accidental ingestion and/or inhalation of small volumes of *Cryptosporidium*-contaminated water. This approach was further supported by the application of robust diagnostic techniques (e.g. immunofluorescence, fluorescence microscopy, modified Kinyoun’s, and modified safranin methylene blue staining), which produced consistent and reproducible results, complemented by PCR and sequencing. Furthermore, sampling sites were selected based on inclusion criteria that strongly indicated potential contamination. The previous findings reflect widespread environmental contamination across interconnected water supplies, likely stemming from untreated human and animal waste. The presence of oocysts in treated tap water suggests systemic failures in water treatment infrastructure or post-treatment contamination. This scenario is particularly alarming given the extremely low infectious dose of *Cryptosporidium* spp., resistance to conventional disinfection methods, prolonged environmental persistence, and the limited availability of reliable diagnostic tools, heightening the risk of large-scale outbreaks, especially among immunocompromised individuals. Furthermore, the mixing of water sources complicates contamination control and points to significant gaps in sanitation, water management, and infrastructure.

The observed prevalence patterns of *Cryptosporidium* in humans, water, and food resources have important implications for public health strategies across the Asian region. The high burden in several countries, particularly where sanitation and water quality control are insufficient, highlights the need for targeted interventions. Enhancing water treatment systems, enforcing stricter food safety protocols, and increasing access to sensitive diagnostic methods such as routine species-level surveillance are essential measures for reducing transmission. Additionally, integrating routine *Cryptosporidium* screening into existing public health surveillance frameworks could facilitate early detection and prompt response to outbreaks. These findings support the development of context-specific policies aimed at mitigating the impact of *Cryptosporidium* spp. on vulnerable populations, especially children and immunocompromised individuals.

## 5 Conclusions

This review provides a comprehensive overview of the prevalence, distribution, and transmission dynamics of *Cryptosporidium* spp. across Asia over the past decade. The findings highlight significant regional/country disparities in infection rates, with Southeast Asia emerging as a hotspot due to its climate, sanitation challenges, and diagnostic limitations. The predominance of *C. hominis* and *C. parvum* across human, water, and food samples indicates a complex interplay of anthroponotic and zoonotic transmission routes. Notably, the detection of alarmingly high oocyst concentrations in drinking and surface water, as well as contamination in fresh produce, underscores the fragility of public health and water safety infrastructures in many Asian countries. Vulnerable groups, particularly immunocompromised individuals, face heightened risks, with prevalence estimates reflecting this concern. The reliance on non-molecular diagnostic methods also points to a critical need for improved surveillance and species-level identification, which can guide future research and interventions. Altogether, these findings call for urgent, coordinated, and multisectoral interventions focused on improving water and food safety, enhancing diagnostic capabilities, and strengthening public health systems to effectively monitor, control, and prevent cryptosporidiosis across the continent.

## Ethical approval

Not applicable.

## CRediT authorship contribution statement

**Shahira Abdelaziz Ali Ahmed:** Data curation, Visualization, Investigation, Validation, Writing - original draft, Writing - review & editing. **Sonia Boughattas:** Data curation, Validation, Visualization, Investigation, Writing - original draft, Writing - review & editing. **Mohammad Reza Mahmoudi:** Data curation, Writing - original draft, Writing - review & editing. **Huma Khan:** Data curation, Writing - original draft, Writing - review & editing. **Simuzar Mamedova:** Data curation, Writing - original draft, Writing - review & editing. **Ardra Namboodiri:** Data curation, Writing - original draft, Writing - review & editing. **Frederick R. Masangkay:** Visualization, Investigation, Data curation, Validation, Writing - review & editing. **Panagiotis Karanis:** Conceptualization, Supervision, Writing - review & editing.

## Funding

This research did not receive any grant from funding agencies in the public, commercial, or not-for-profit sectors.

## Declaration of competing interests

The authors declare that they have no known competing financial interests or personal relationships that could have appeared to influence the work reported in this paper.

## Data Availability

The data supporting the conclusions of this article are included within the article and its supplementary files.
